# Calibration of discrete element parameters for cohesive soils at different moisture contents

**DOI:** 10.1371/journal.pone.0340462

**Published:** 2026-02-09

**Authors:** Shixi Wei, Bing Li, Jinxia Yang, Dengsheng Cai, Wubin Xu, Zhaoyang Liu

**Affiliations:** 1 School of Mechanical and Automotive Engineering, Guangxi University of Science and Technology, Liuzhou, China; 2 Guangxi Colleges and Universities Engineering Research Center for High-End Engineering Machinery Equipment, Liuzhou, China; 3 Guangxi Zhuang Autonomous Region Engineering Research Center for Intelligent Manufacturing of High-End Engineering Equipment, Liuzhou, China; 4 Guangxi Liugong Machinery Co., Ltd., Liuzhou, China; Jouf University, SAUDI ARABIA

## Abstract

To obtain discrete element method simulation parameters for cohesive soil during the scrapping process of loaders, this paper calibrates parameters of cohesive soil with different moisture contents based on the Hertz-Mindlin with Johnson-Kendall-Roberts (JKR) Cohesion contact model in Experts in Discrete Element Modeling (EDEM). First, five cohesive soil samples with different moisture contents were prepared. By combining vibration sieving, the inclined plane method, and angle of repose experiments, the measured data ranges of particle size distribution, soil-steel friction coefficient, and angle of repose were obtained. Secondly, the JKR V2 adhesion model was constructed in EDEM. Significant parameters were screened using the Plackett-Burman test. Finally, the response surface analysis range was determined by integrating climbing experiments, and a quadratic regression model was established through Box-Behnken design to optimize parameter combinations. Furthermore, a Particle Swarm Optimization (PSO) algorithm was introduced for single-objective optimization of the angle of repose. The experimental results show that with the increase of moisture content, the angle of repose increases from 30.83° to 37.13°, and the significant parameters are JKR surface energy, soil-soil restitution coefficient, and static friction coefficient. The simulation error of the PSO algorithm is reduced from the maximum 3.38% in the response surface method to within 2.2%. This study provides a high-precision parameterization method for DEM modeling of cohesive soil, offering references for establishing DEM simulations of loaders scraping cohesive soil.

## 1. Introduction

The scrapping resistance of loaders is a key indicator for measuring scrapping performance. In earthwork, mining, and other operations, the moisture content of cohesive soil significantly affects its cohesion and scrapping resistance, so studying the mathematical model under different moisture contents is of great significance. Existing studies mostly focus on non-cohesive bulk materials such as fine sand and crushed stone. The analysis of cohesive soil is limited to a single moisture content, and simulation models often simplify factors such as humidity and adhesiveness, leading to large deviations from actual working conditions. The Discrete Element Method can efficiently shorten the design cycle, so this study focuses on low-moisture-content cohesive soil to establish a discrete element model considering adhesive force for accurate simulation of the actual scrapping process.

In the research field of soil material handling by loader buckets, several studies have been conducted. Nezami [1] proposed a discrete element simulation with polyhedral particles, primarily investigating the influence of shape on modeling large soil particles. Abbas Hemmat [2] developed a single-tip and multi-tip horizontal permeameter for measuring soil mechanical resistance, focusing on continuous medium soils. TAKAHASHI [3] performed resistance analysis on buckets based on soil mechanics principles. Kenji Tsuchiya [4] studied soil deformations and estimated resistance through their deformed shapes. Siddharth Dadhich [5] and Stéphane Blouin et al [6] analyzed continuous medium soils. Sarata [7] used Coulomb’s earth pressure theory to investigate the relationship between resistance, bucket movement, and cohesionless soil. Dong-Kwan Seo [8] developed a soil model through mechanical soil analysis, establishing a theoretical framework for subsequent research. Koji Aoshima [9] determined the impact of gravel soil particle size on loading resistance through discrete element simulation and experimental validation, analyzing the gap between simulation and reality. Bingwei Cao et al [10] studied an intelligent control method for loader buckets by observing manual loading operations on soil piles. Bobbie Frank et al [11] analyzed the relationship between resistance and trajectory for crushed stone materials, seeking optimal loading trajectories through repeated experiments.

Discrete Element Modeling is a numerical method for modeling the mechanical behavior of granular materials [12]. Fubin Zhang [13] conducted a bionic design to establish a JKR model for wet and sticky soils in rice-wheat rotation areas with uniform moisture content. Yuan Wan [14] measured physical parameters of Hami melon seeds, calibrated DEM simulation parameters, obtained optimal parameter combinations, and validated model reliability, providing a theoretical basis for seed metering device design and optimization. This method verified its feasibility. Shengsheng Wang [15] researched physical parameters of Chinese cabbage seeds, calibrated DEM simulation parameters through multiple tests, obtained optimal combinations with a validation error of 0.73%, providing parameters for mechanized harvesting. Zhou Tienan [16] calibrated DEM model parameters for soaked paddy field soil based on slump tests, obtained optimal combinations with a 2.04% validation error, providing a basis for paddy field puddling simulation. Tao Chen [17] calibrated contact parameters for DEM simulation of alfalfa stems at the early flowering stage through physical tests and multiple simulations, obtained optimal combinations with a 0.48% validation error, providing parameter support for forage machinery research. Lintao Chen [18] proposed a method for calibrating DEM parameters of bamboo powder based on dimensional analysis and BP neural network, which showed an error of less than 2.3% through tests, providing a reference for related equipment development. SONG Zhan-hua [19] studied DEM model parameters for mulberry orchard soil, using the Hertz-Mindlin with Bonding contact model. Jianzhong Zhu [20] investigated contact parameters for DEM simulation of lunar soil, determining the contact parameters between lunar wheels and soil. When modeling soil with DEM, continuous JKR models are commonly used, but during loader bucket operations, the material is mostly discontinuous granular matter; JKR values vary in this context.

Efficient loader loading of cohesive soil and control of equipment wear in earthwork and mining operations rely on accurate quantification of its mechanical behavior. Traditional research faces bottlenecks, and the introduction of the discrete element method (DEM) is critical, with its necessity reflected in three aspects: First, traditional continuous medium models fail to capture the microscopic dynamic behavior of cohesive soil, resulting in large errors in loading resistance and inability to explain the cohesion variation mechanism, whereas DEM quantifies the correlation between microscopic parameters and macro responses via discrete particles. Second, traditional physical tests struggle to reproduce the multi-stage dynamic loading process, while DEM can construct a 1:1 simulation model to replicate mechanical responses and adjust parameters—an advantage unmatched by traditional methods—providing a dynamic basis for optimizing operational parameters. Third, traditional trial-and-error parameter calibration is inefficient with limited applicability, while DEM combined with intelligent optimization methods can quickly identify significant parameters, improving calibration efficiency by 3–5 times and reducing repose angle simulation errors to support engineering practice. Thus, this study adopts the DEM.

Among the cohesive contact models in EDEM software, the Linear Cohesion Model (empirical constant cohesion, incompatible with dynamic water content changes) and Hertz-Mindlin with Bonding Model (virtual bonds, unable to simulate reversible cohesion) both have mechanism or adaptability flaws. In contrast, the JKR V2 Model-based on the coupled mechanism of surface energy and liquid bridge force-features parameters with clear physical significance, can dynamically respond to water content changes and adapt to the reversible cohesion of cohesive soil, and is comprehensively optimal in mechanism authenticity, adaptability, and engineering efficiency. Thus, this study selects it for simulating cohesive soil.

This paper carries out discrete element parameter calibration experiments for cohesive soil at different moisture contents, using the JKR V2 model in adhesive contact for modeling and simulation of cohesive soil particles. The contact model requires determining the intrinsic parameters of cohesive soil and steel, obtaining the soil-steel friction coefficient through experiments, and acquiring the range of other parameters through GEMM. Plackett-Burman tests were used to screen significant parameters, climbing experiments determined the response surface analysis range, Box-Behnken design established a quadratic regression model to optimize parameter combinations, and a Particle Swarm Optimization (PSO) algorithm was further introduced for single-objective optimization of the angle of repose.

Compared with the advanced calibration technology for ball milling [21], it shares consistent parameter screening logic and multi-scale correlation goals. This study adds “water content-parameter sensitivity” analysis for multi-condition adaptability, while the latter focuses on a single material state.

Compared with the novel coarse-grained DEM method for bonded materials [22], both focus on DEM parameter calibration, adopting the workflow of “experimental measurement - parameter screening - response surface optimization - model verification” and incorporating Plackett-Burman design/steepest ascent method, with minimum error as the optimization goal, extended contact mechanics models, and validation by physical experiments and simulations. The difference is that this study integrates PSO optimization for higher accuracy; future work may involve a crop-soil coupling system for standardization and refinement.

Compared with the application of statistical design optimization in geotechnical engineering [23], Share consistent methodology and complementary contact models, with physical experiment indicators as benchmarks to verify the validation system’s versatility across agricultural biomaterials and geotechnical bulk materials.

Compared with the fractional elastoplastic constitutive model [24], both target “wide-graded and high-water-content” soils, adopt the same calibration chain, and align on experimental design and error control. This study focuses on the positive correlation between water content, JKR surface energy, and angle of repose (static accumulation), while the latter is embedded in the CFD-DEM framework to verify multiple indicators (seepage-migration coupling), forming complementary “wet-dry mechanics and seepage-erosion mechanics” parameter libraries.

**Fig 1:**
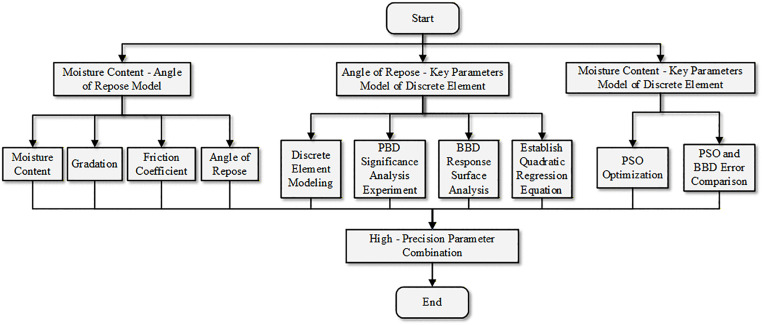
Flow chart of discrete element parameter calibration for cohesive soil under different water contents.

The accuracy of the model was analyzed using error values. The specific flow chart is shown in Fig 1.

## 2 Materials and methods

### 2.1 Intrinsic parameters of cohesive soil

**Fig 2:**
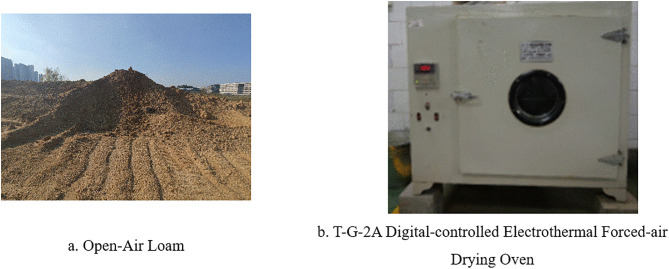
Materials and equipment.


The cohesive soil was taken from southern China, as shown in Fig 2A. To study the discrete element key parameters of the angle of repose formed by water content when treating it as bulk material, the cohesive soil was dried using a T-G-2A digital electrothermal constant-temperature forced-air drying oven (Fig 2B), and pure water was added to prepare five cohesive soil samples with different moisture contents. The calculation method is:

$$w=\frac{m_{w}}{m_{2}}\times 100\%=\frac{m_{1}-m_{2}}{m_{2}}\times 100\%$$ (1)

where *w* is the soil moisture content,%; *m*_*w*_ is the specific gravity of soil particles, g; *m*_*1*_ is the weight of soil before drying, g; and *m*_*2*_ is the weight of soil after drying, g.

Five cohesive soil samples with moisture contents of 0%, 5%, 10%, 15%, and 20% were prepared using Eq 1. Density is a measure of mass in a specific volume, and in DEM simulation technology, it is the dry density of materials, calculated as:


$$\rho_{s}=\frac{m_{s}}{V_{s}}$$ (2)

where *ρ*_s_ is the mass of water in the soil, kg/m^3^; *m*_s_ is the ratio of soil particle mass, kg; *V*_s_ is the weight of soil before drying,m^3^.

The measured dry densities of the soil were 2384 kg/m^3^, 1980 kg/m^3^, 1802 kg/m^3^, 1653 kg/m^3^, and 1527 kg/m^3^ in sequence. When conducting DEM simulations for materials with different moisture contents, the intrinsic parameters adopt average values. Thus, the shear modulus of cohesive soil with a moisture content of 10% ± 1% is 1 × 10^6^ Pa, and Poisson’s ratio is 0.38 [25].

### 2.2 Particle size distribution of cohesive soil

**Fig 3:**
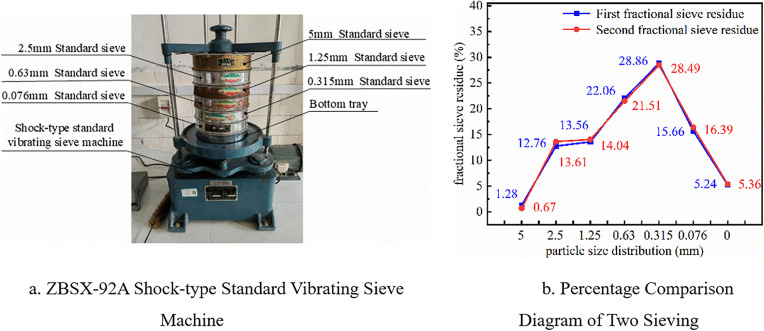
Particle sieving experiment.

The vibration sieving method was used to distribute the particle sizes of dry soil. A set of standard sieves with different apertures was used to place air-dried and dispersed representative samples into a ZBSX-92A shock-type standard vibrating sieve (Fig 3A). The weight of dry soil retained on each sieve was weighed, and the relative content of each particle group was calculated to determine the particle size distribution of the soil from the particle analysis results. The sieve apertures were 5 mm, 2.5 mm, 1.25 mm, 0.63 mm, 0.315 mm, and 0.076 mm. The samples were divided into four parts using the quartering method, and two diagonal samples were taken for parallel comparative experiments. The experimental data are shown in Fig 3B.

The results show that the errors of the two experiments are both less than 1% of the initial mass, meeting the experimental requirements. The small-mass particles were removed, and the large particles were retained for simulation. It is specifically stipulated that the percentage of particle size distribution of 2.5−5 mm is 14%, 1.25–2.5 mm is 14%, 0.63–1.25 mm is 22%, and 0.315–0.63 mm is 50%.

### 2.3 Angle of repose experiment for cohesive soil

The angle of repose experiment is used to measure the maximum slope angle when bulk materials such as particles, powders, and cohesive soil naturally accumulate into a stable cone. This angle reflects the friction, adhesive force, and particle size characteristics of the material: a smaller angle indicates better fluidity (e.g., fine sand), and a larger angle indicates poorer fluidity (e.g., wet clay). The stability of material accumulation is determined by friction and adhesive force between particles.

**Fig 4:**
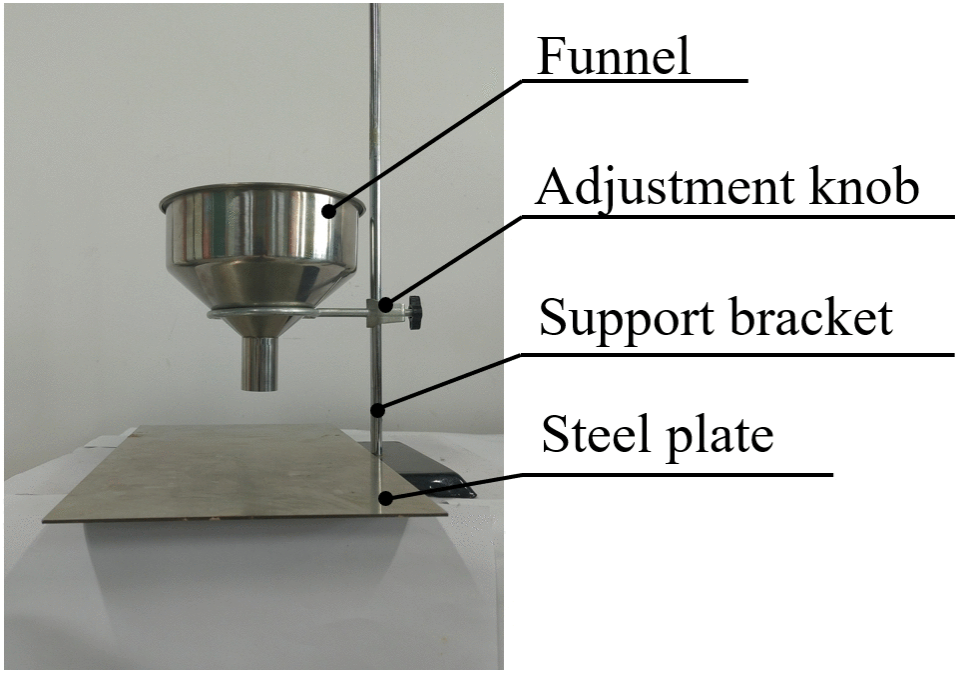
Experiment on the angle of repose of soil.

Experimental method and device: This paper uses a funnel experimental device with a top diameter of 150 mm, a bottom diameter of 30 mm, a height of 130 mm, and a distance of 70 mm between the bottom of the funnel and the receiving plate, as shown in Fig 4.

**Fig 5:**
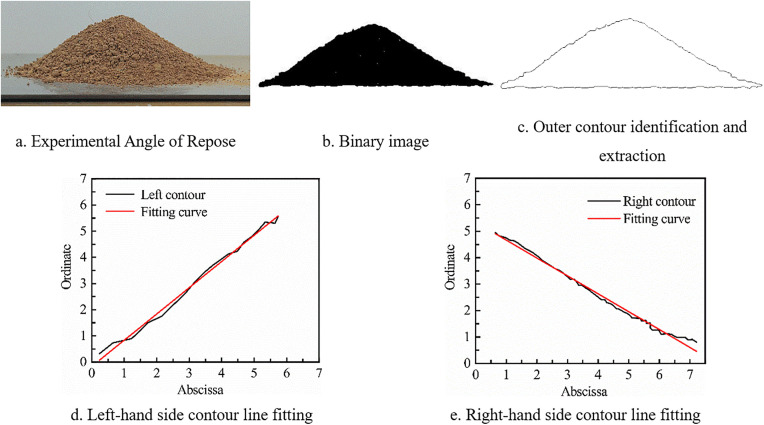
Schematic diagram of the process of extracting and fitting contour curves in MATLAB.

Experimental steps: Each time, 200–250 g of soil was taken and placed in the funnel. After covering the bottom, the soil was allowed to fall freely to form a pile. The particle pile image was extracted separately, binarized using MATLAB, the edge image was extracted, and the contour lines on both sides were fitted. The process is shown in Fig 5.

As shown in the experimental procedure of Fig 5, the angle of repose experiments were conducted on cohesive soils with different moisture contents. The experimental data were measured ten times, and the highest and lowest values were removed. The remaining values were averaged, and the data were sorted as shown in Table 1. It is concluded that the angle of repose increases with the increase in moisture content.

**Table 1:** Angle of repose of cohesive soils with different moisture contents.

Moisture content/%	Experimental value of angle of repose/°
0	30.83
5	31.56
10	31.97
15	33.15
20	37.13

**Fig 6:**
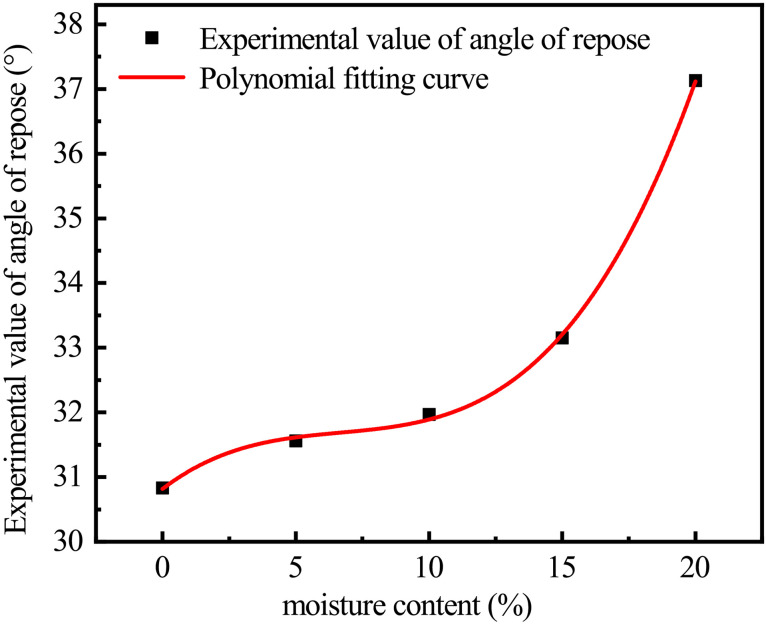
Regression model of moisture content-angle of repose for cohesive soil.


The results in Table 1 were subjected to polynomial fitting as shown in Fig 6, and a polynomial regression equation model of moisture content-angle of repose for cohesive soil was established as shown in Eq 3. The residual sum of squares of the model was 0.01262, and the R² was 0.9995, indicating a very high correlation coefficient of the model.

$$Y=30.8166+0.3156X-0.0416X^{2}+0.00208X^{3}$$ (3)

Static Friction Coefficient and Liquid Bridge/Capillary Force: At low water content, liquid bridges are scarce, and the static friction between particles is mainly generated by contact via surface roughness, resulting in a low coefficient. As water content increases, the number of liquid bridges rises, and the “bonding effect” they induce enhances the relative sliding resistance between particles, leading to an increase in the static friction coefficient. When water content is excessively high, the excess water exerts a lubricating effect, offsetting part of the bonding force, and the static friction coefficient slightly decreases instead [26].

JKR Surface Energy and Liquid Bridge/Capillary Force: At low water content, the water film on the particle surface is thin, liquid bridges are scarce and weak, and the JKR surface energy (characterizing the particle adhesion capacity) is relatively small. With the increase in water content, the water film thickens, and a large number of liquid bridges form; the tension of the liquid bridges is converted into the adhesion force between particles, causing the JKR surface energy to increase accordingly. When water content is excessively high, liquid bridges fuse into a continuous water body, capillary force weakens, and the JKR surface energy tends to stabilize or slightly decrease.

### 2.4 Measurement of friction coefficient range

**Fig 7:**
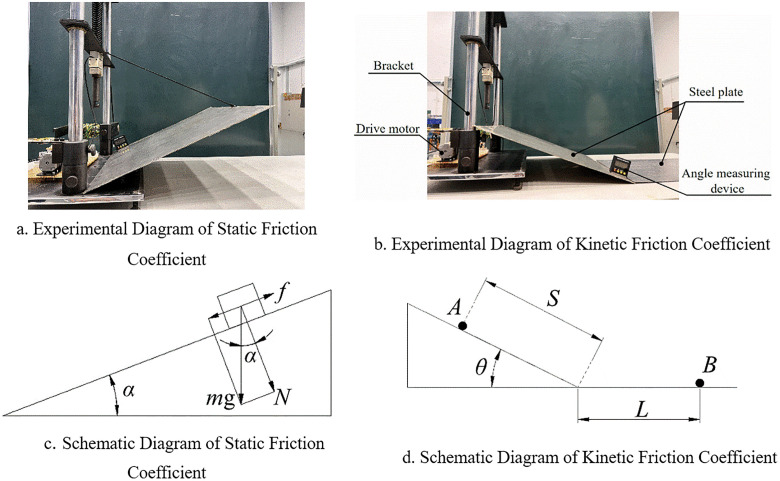
Experiment for measuring static and dynamic friction coefficients.


The inclined plane method [27] was used to measure the static friction coefficient. As shown in Fig 7A, the material was placed on an inclined steel plate, the inclined angle was adjusted until the material just slid down, and the inclined angle was recorded. Ten groups of experiments were conducted for each moisture content to retain the range of coefficient values. For the rolling friction coefficient experiment [27], as shown in Fig 7B, a soil ball was placed at a fixed angle (30°) and allowed to roll freely without external force. The distance *S* was 100 mm, and the value of *L* was recorded, where *A* is the initial position of the ball, and *B* is the final stop position of the ball. The calculation formulas for the static and rolling friction coefficients are shown in Eq 7 and Eq 8. Ten groups of experiments were conducted for the static and dynamic friction coefficients of cohesive soil at different moisture contents, and the experimental data are shown in Table 2.

**Table 2:** Static and dynamic friction coefficients of cohesive soil at different moisture contents.

Moisture content	0	5%	10%	15%	20%
Soil-Steel Static Friction Coefficient	0.36-0.43	0.34-0.57	0.35-0.45	0.31-0.47	0.51-0.68
Soil-Steel Dynamic Friction Coefficient	0.248-0.33	0.4-0.5	0.35-0.45	0.16-0.2	0.15-0.2

f=F=mgsinα (4)

N=mgcosα (5)

$$f=\mu_{s}N$$ (6)

$$\mu_{s}=\frac{f}{N}=\frac{mg\sin\alpha }{mg\cos\alpha }=\tan\alpha$$ (7)

$$\mu_{f}=\frac{S\times \sin\theta }{S\times \cos\theta +L}$$ (8)

Where *f* is the static friction force, in Newtons, N; *α* is the static friction angle of the inclined plane, in degrees (°); *N* is the normal pressure, in Newtons, N; *μ*_*s*_ is the static friction coefficient; *μ*_*f*_ is the dynamic friction coefficient; *S* is the length of the spherical soil on the inclined plane, in millimeters, mm; *θ* is the fixed angle of the inclined plane, in degrees (°); *L* is the rolling distance of the spherical soil on the horizontal plate, in millimeters, mm;

### 2.5 Acquisition of GEMM parameters

Using the obtained soil density, angle of repose, and other values, the soil-soil static friction coefficient, soil-soil rolling friction coefficient, soil-soil restitution coefficient, soil-steel restitution coefficient, and JKR surface energy were selected from the GEMM library. The values are shown in Table 3.

**Table 3:** Recommended range of parameter values in the GEMM library.

Parameters	0%	5%	10%	15%	20%
Soil-soil restitution coefficient	0.15-0.75	0.15-0.75	0.15-0.75	0.15-0.75	0.35-0.75
Soil-soil static friction coefficient	0.2-1.16	0.2-1.04	0.32-1.16	0.2-1.16	0.2-1.04
Soil-soil rolling friction coefficient	0-0.2	0-0.05	0-0.05	0-0.2	0.05-0.2
Soil-steel restitution coefficient	0.15-0.75	0.15-0.75	0.15-0.75	0.15-0.75	0.15-0.75
JKR surface energy/ J·M ⁻ ²	0-18.75	0-25	0-18.75	0-25	0-25

### 2.6 Discrete element EDEM modeling

Through the preliminary experiments, all simulation parameters of the JKR V2 contact model were obtained. In this paper, the intrinsic parameters of cohesive soil particles and steel were set as fixed average values to explore the significant influence of different parameters on the angle of repose, and then generalized to the angle of repose values at different moisture contents. The steel used in the experiment has the following intrinsic parameters: solid density of 7850 kg/ m³ [25], Poisson’s ratio of 0.3, and shear modulus of 7.9 × 10⁴ MPa [28].

**Fig 8:**
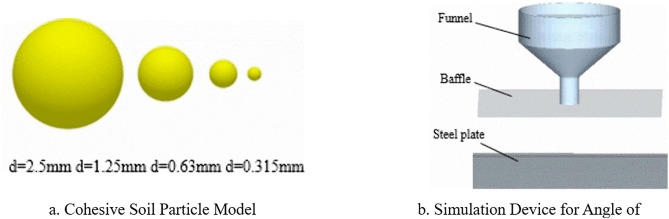
EDEM model. **a.** Cohesive Soil Particle Model. **b.** Simulation Device for Angle of.

The particle model is shown in Fig 8A, with particle sizes and percentages of 2.5 mm (14%), 1.25 mm (14%), 0.63 mm (22%), and 0.315 mm (50%). The 1:1 model for the angle of repose simulation is shown in Fig 8B. The simulation settings were a total generated mass of 0.2 kg, a generation rate of 0.05 kg/s, a fixed simulation time step of 20%, a data saving interval of 0.01 s, and a total simulation duration of 10 s.

## 3 Results and discussion

### 3.1 Plackett-Burman (PBD) significance test analysis

The Plackett-Burman (PBD) test is an efficient screening test method based on non-orthogonal balanced design theory. It evaluates the main effects of multiple factors on the response variable in less test time. In this paper, 12 test times were used for 7 factors. This method is suitable for quickly screening key factors from many potential influencing factors. The test sets each factor to two levels: low (−1) and high (+1), uses a specially designed matrix to ensure uniform distribution of factor level combinations, and judges the influence degree of factors through effect estimation and significance testing, mainly analyzing main effects without considering interactions to reduce test costs and improve research efficiency. The range values of other parameters determined above are sorted as shown in Table 4, with symbols and parameter values used as the level inputs for the PB experiment.

**Table 4:** Parameter ranges of JKR V2 model for cohesive soil and steel.

Contact parameters	Symbol	Level	
		Low level	High level
Soil-Soil Restitution Coefficient	*A*	0.15	0.75
Soil-Soil Static Friction Coefficient	*B*	0.2	1.16
Soil-Soil Rolling Friction Coefficient	*C*	0	0.2
Soil-Steel Restitution Coefficient	*D*	0.15	0.75
Soil-Steel Static Friction Coefficient	*E*	0.36	0.68
Soil-Steel Dynamic Friction Coefficient	*F*	0.15	0.43
JKR Surface Energy/J·M^-2^	*G*	0	10

Among the parameter recommendation ranges in the GEMM library, the soil-soil restitution coefficient, static/rolling friction coefficients, soil-steel restitution coefficient, and JKR surface energy are referenced. Except for JKR surface energy, other parameters adopt library values to avoid losing sensitivity due to an excessively narrow range. JKR surface energy is adjusted from 0–25–0–10 J·m ⁻ ²: preliminary experiments show the repose angle error reaches nearly 100% at JKR = 10 and approaches 90° at JKR = 25. This adjustment avoids parameter dilution and interference with the P-B test caused by an overly wide range (ensuring accurate capture of G’s significance) while preventing sensitivity loss from an excessively narrow range.

Based on the parameters in Table 4, PBD experiments were conducted, and significance analysis was carried out using Design-Expert 13.0 software by inputting the measured results of the angle of repose. The experimental scheme and results are shown in Table 5, where the angle of repose was analyzed using the aforementioned MATLAB method for contour curve fitting.

**Table 5:** Scheme and results of Plackett-Burman design.

Serial number	Symbols							Angle of repose/°
	*A*	*B*	*C*	*D*	*E*	*F*	*G*	
1	−1	1	−1	1	1	−1	1	38.7
2	−1	1	1	−1	1	1	1	31.74
3	−1	−1	1	−1	1	1	−1	13.94
4	1	−1	1	1	1	−1	−1	18.36
5	1	−1	1	1	−1	1	1	85.29
6	−1	−1	−1	−1	−1	−1	−1	14.93
7	−1	−1	−1	1	−1	1	1	76.73
8	−1	1	1	1	−1	−1	−1	17.47
9	1	1	−1	1	1	1	−1	22.52
10	1	1	1	−1	−1	−1	1	56.06
11	1	−1	−1	−1	1	−1	1	74.87
12	1	1	−1	−1	−1	1	−1	26.68

**Fig 9:**
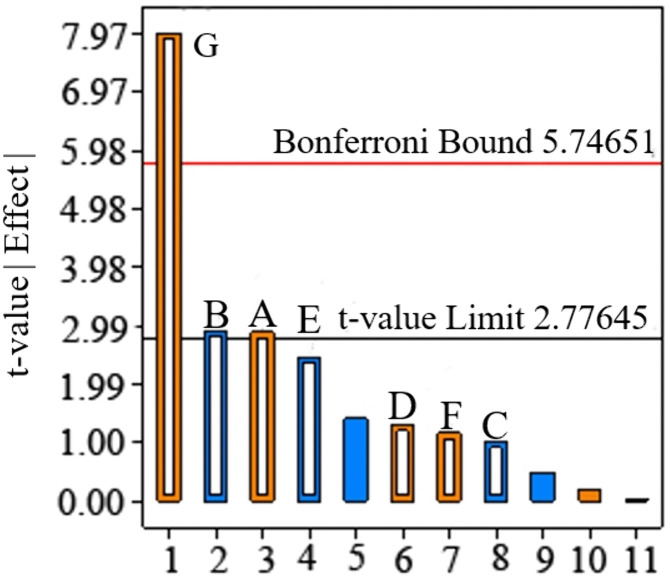
PB Pareto chart.

As shown in Table 6 and Fig 9, the model is significant. The G factor (JKR surface energy) has an extremely significant influence on the angle of repose, while the A and B factors (soil-soil restitution coefficient and soil-soil static friction coefficient) have significant influences, with all three factors having values above 2.77. The model has 7 degrees of freedom without interaction, and the F-value of 12.91 indicates that the model is significant. The probability of such a large F-value occurring due to noise is only 1.32%.

**Table 6:** Significance analysis.

Source	Sum of squares	df	Mean square	*F*	*P*
Model	7383.15	7	1054.74	12.91	0.0132*
*A*	679.06	1	679.06	8.31	0.0449*
*B*	689.33	1	689.33	8.44	0.0439*
*C*	83.06	1	83.06	1.02	0.3703
*D*	139.06	1	139.06	1.7	0.262
*E*	494.47	1	494.47	6.05	0.0697
*F*	111.08	1	111.08	1.36	0.3084
*G*	5187.11	1	5187.11	63.5	0.0013**
Residual	326.76	4	81.69	–	–
Cor Total	7709.91	11	–	–	–

Note: ** indicates extremely significant (P < 0.01), and * indicates significant (P < 0.05). The same applies hereinafter.

Table 6 and Fig 9 show the model is significant (F = 12.91, p = 0.0132), verifying the reliability of test results. JKR surface energy (G, F = 63.5, p < 0.01) is extremely significant, as the dominant factor affecting the angle of repose, verifying the rationality of the JKR V2 model. Soil-soil restitution coefficient (A, F = 8.31) and static friction coefficient (B, F = 8.44) are significant (p < 0.05), jointly regulating soil stacking balance with moisture-dependent sensitivity. Insignificant parameters (p > 0.05), such as soil-soil rolling friction coefficient (C, F = 1.02) and soil-steel restitution coefficient (D, F = 1.7) have a negligible impact on the angle of repose, facilitating invalid parameter exclusion.

Extreme angle of repose values in Table 5 are boundary effects of parameter combinations, confirming the PBD test covers extreme scenarios and ensures the full-range regulatory effect of significant parameters.

### 3.2 Climbing experiment

Based on the above experiments, *G*, *A*, and *B* were identified as the main climbing objects. The climbing experiment (Ramp Test) is a method to determine the optimal process parameter range or critical threshold by gradually and continuously adjusting the levels of independent variables and following the response trend of dependent variables. It is characterized by gradualness, changing variables at a fixed step size, exploring laws of unknown systems, and outlining trends through a small number of experiments, widely used in chemical engineering, materials science, environmental engineering, biomedical, and other fields. By setting a reasonable step size for gradient descent search, the error analysis was conducted using the experimental angle of repose value (31.97°) of cohesive soil with 10% moisture content as the standard value and the simulation value. The error *Ψ* calculation formula:


$$\psi =\frac{\left|Y-Y^{\prime }\right|}{Y^{\prime }}\times 100\%$$ (9)

where *Y* is the simulated value of the angle of repose (°); *Y’* is the experimental value of the angle of repose (°).

According to Table 7, the error value is the smallest at Serial Number 3, and the overall trend shows a decrease first and then an increase. This proves that the ideal value can be achieved near Serial Number 3. Therefore, in the subsequent response surface analysis, Serial Numbers 2, 3, and 4 were used as the low, medium, and high levels for experiments. When the data reached Serial Numbers 9 and 10, the error exceeded 100%, indicating that the three factors had a significant influence on the model.

**Table 7:** Steepest ascent experiment and error analysis.

Serial number	*A*	*B*	*G*	*Y*/°	*Ψ*/%
0	0.15	0.2	0	14.71	53.99
1	0.21	0.296	0.02	20.12	37.08
2	0.27	0.392	0.04	22.18	30.63
3	0.33	0.488	0.06	30.12	5.77
4	0.39	0.584	0.08	45.05	40.93
5	0.45	0.68	0.1	48.63	52.10
6	0.51	0.776	0.12	54.61	70.82
7	0.57	0.872	0.14	60.04	87.80
8	0.63	0.968	0.16	62.89	96.73
9	0.69	1.064	0.18	67.67	111.66
10	0.75	1.16	0.2	72.32	126.21

Error decrease stage (Serial Number 0–3): As parameters increase from low levels to medium levels, the adhesion (characterized by G) and sliding resistance (characterized by B) between particles gradually match the actual mechanical properties of cohesive soil, so the simulation value of the angle of repose is close to the experimental value, and the error decreases rapidly.

Error increase stage (Serial Number 3–10): When parameters exceed the medium level, excessive G leads to excessive adhesion between particles (forming “rigid aggregates” that cannot slide freely), and excessive B leads to excessive sliding resistance. As a result, the simulated angle of repose is much larger than the actual value, and the error increases sharply (even exceeding 100% at No. 9–10), indicating that the three factors have a significant impact on the model.

The climbing experiment determines that the range of response surface analysis is the levels corresponding to Serial Number 2−4 (A: 0.27–0.39, B: 0.392–0.584, G: 0.04–0.08). This range not only covers the optimal parameter interval but also reserves sufficient gradients to fit the quadratic regression model, avoiding model underfitting caused by too narrow a range. At the same time, the V-shaped error trend also confirms the synergistic effect between A, B, and G—adjusting a single parameter cannot minimize the error, which provides a basis for introducing quadratic terms (such as B², G²) in subsequent response surface analysis.

### 3.3 Response surface analysis

The Box-Behnken design (BBD) is a common three-level factor experimental design technique in the response surface method (RSM), often used to fit the relationship between variables and independent variables, establish a quadratic regression model, and analyze their interactions. This analysis method codes variables into high, medium, and low three levels. The climbing sequence numbers 2, 3, and 4 were coded as −1, 0, and 1 levels for experiments, with a total of 17 groups of experiments and 5 central point repeated experiments. The experimental scheme and results are shown in Table 8. The mathematical model between the angle of repose and *A*, *B*, and *G* is shown in Eq 10.

**Table 8:** Box-Behnken experimental design and results.

Serial number	*A*	*B*	*G*	*Y*
1	0	0	0	29.7425
2	1	0	1	43.6547
3	1	0	−1	28.4294
4	1	1	0	36.8031
5	−1	−1	0	28.1367
6	0	0	0	30.0755
7	0	−1	1	42.7364
8	0	1	1	45.0587
9	0	1	−1	27.9500
10	0	−1	−1	23.9867
11	−1	1	0	34.5371
12	−1	0	1	40.1627
13	0	0	0	30.1615
14	0	0	0	28.074
15	0	0	0	29.0421
16	−1	0	−1	23.4424
17	1	−1	0	32.1128


$$Y=\;29\mathrm{.42}+1\mathrm{.84}A+2\mathrm{.17}B+8\mathrm{.48}G-0\mathrm{.4275}AB-0\mathrm{.3738}AG-0\mathrm{.4103}BG+1\mathrm{.23}A^{\text{2}}\;+\;2\mathrm{.24}B^{\text{2}}\;+\;3\mathrm{.27}G^{\text{2}}$$ (10)

The analysis of variance (ANOVA) for the BBD test results is shown in Table 9. The model has a P-value < 0.0001 and a lack-of-fit term of 0.2104 (which is > 0.05), indicating the model is extremely significant overall, and the lack of fit is non-significant, effectively describing the relationship between variables. The P-values of *A*, *B*, and *G* are all < 0.05, showing that the main effects of *A*, *B*, and *G* significantly influence *Y*, with *G* being extremely significant. The quadratic terms *B²* and *G²* exhibit significant nonlinear influences, indicating that the curvature effects of factors *B* and *G* and their interaction prominently affect the response results. The model has a pure error of 0.7595, an *R²* of 0.9884, and an adjusted *R²* of 0.9735, providing a reliable basis for subsequent parameter optimization. The level combinations of significant factors and their nonlinear relationships require focused attention.

**Table 9:** Box-Behnken Analysis of Variance (ANOVA).

Source	Sum of squares	*df*	Mean square	*F*	*P*
Model	721.17	9	80.13	66.28	< 0.0001^**^
*A*	27.09	1	27.09	22.41	0.0021^*^
*B*	37.74	1	37.74	31.22	0.0008^*^
*G*	574.67	1	574.67	475.31	< 0.0001^**^
*AB*	0.7311	1	0.7311	0.6047	0.4623
*AG*	0.5588	1	0.5588	0.4621	0.5185
*BG*	0.6732	1	0.6732	0.5568	0.4799
*A²*	6.41	1	6.41	5.3	0.0548
*B²*	21.21	1	21.21	17.54	0.0041^*^
*G²*	45	1	45	37.22	0.0005^*^
Residual	8.46	7	1.21	–	–
Lack of Fit	5.43	3	1.81	2.38	0.2104
Pure Error	3.04	4	0.7595	–	–
Cor Total	729.64	16	–	–	–

**Fig 10:**
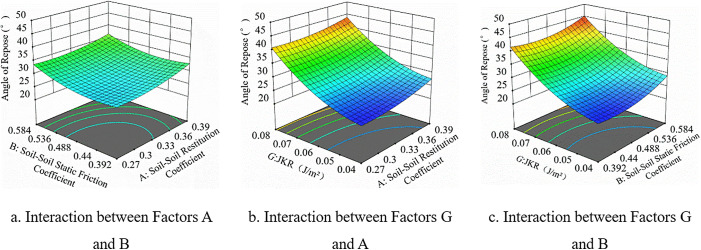
Response surface interaction analysis plots.

Based on the interaction analysis in Fig 10. When *G* (JKR value) is fixed, the angle of repose is less affected by the interaction between *A* and *B*, showing an overall trend of first decreasing and then increasing in Fig 10A. Fig 10B and Fig 10C are highly similar, indicating that the interactions between *A* (soil-soil restitution coefficient), *B* (soil-soil static friction coefficient), and *G* are similar. When their values are kept constant, the response value decreases with the decrease of *G*. When *G* is fixed, the response value increases with the increase of *A* and *B*, indicating that both have a positive correlation with the angle of repose.

### 3.4 Model construction of moisture content and key discrete element parameters

By linking the moisture content-angle of repose model and the angle of repose-discrete element key parameter model for cohesive soil, the moisture content-discrete element key parameter model for cohesive soil was constructed, as shown in Eq 11.


$$\begin{array}{c} 30.8166+0.3156X-0.0416X^{2}+0.00208X^{3}=29\mathrm{.42}+1\mathrm{.84}A+2\mathrm{.17}B+8\mathrm{.48}G\\ -0\mathrm{.4275}AB-0\mathrm{.3738}AG-0\mathrm{.4103}BG+1\mathrm{.23}A^{\text{2}}+2\mathrm{.24}B^{\text{2}}+3\mathrm{.27}G^{\text{2}} \end{array}$$ (11)

The model between the moisture content of cohesive soil and the key discrete element parameters was used to optimize the parameters of the angle of repose of cohesive soil at different moisture contents. 100 groups of optimizations were carried out for each moisture content, and the optimal groups and their simulation results are shown in Table 10.

**Table 10:** BBD optimization of regression equation and its simulation results.

Moisture content /%	*A*	*B*	*G*	*Y*/°
0	0.283	0.464	0.066	30.39
5	0.286	0.409	0.066	31.97
10	0.322	0.579	0.068	31.94
15	0.303	0.581	0.07	32.36
20	0.281	0.417	0.072	38.39

### 3.5 Single-Objective Optimization Algorithm (PSO)

The Particle Swarm Optimization (PSO) algorithm is a meta-heuristic optimization algorithm that simulates the collective cooperation behavior of bird flocks or fish schools, solving the optimal solution for single-objective continuous optimization problems through iterative search. It continues iterating until preset termination conditions (maximum number of iterations or precision requirements) are met, finally outputting an approximate optimal solution. In the algorithm, the number of particles is set to 100, the number of iterations is 1000, the individual learning factor and social learning factor are both 1.8, and the inertia weight ranges from a minimum of 0.2 to a maximum of 0.9. The selection basis is as follows: For the acceleration coefficients, symmetric values are adopted to balance the particle’s exploration of its own historical optimal solution and utilization of the global optimal solution of the swarm, avoiding local optima caused by one-way search and adapting to the 3D parameter optimization scenario. For the dynamic inertia weight, an initial value of 0.9 ensures a wide global search range, and it linearly decreases to 0.2 during iterations to enhance local refined search, solving the problem that fixed weights are difficult to balance “optimization breadth and precision”. For the number of particles and iterations: 100 particles cover key regions of the parameter space, and 1000 iterations reserve sufficient convergence time.

**Fig 11:**
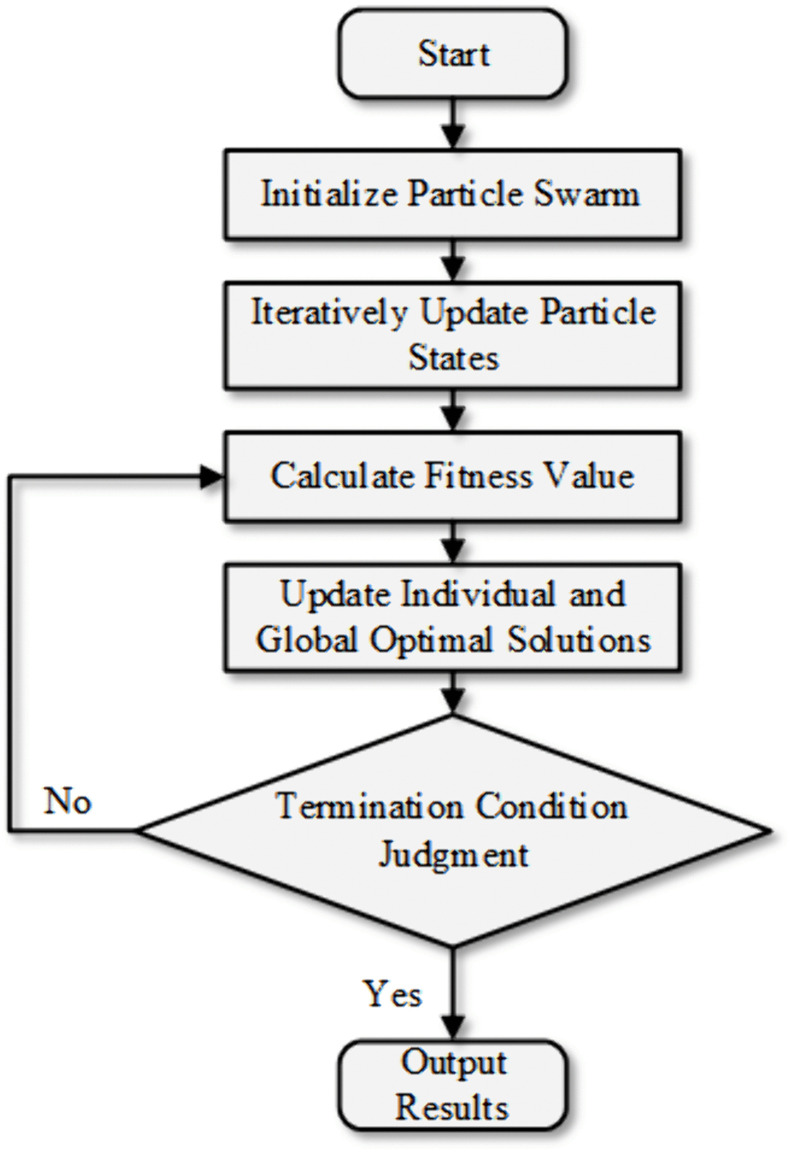
Flowchart of Particle Swarm Optimization (PSO).

The algorithm flow chart is shown in Fig 11.

The BBD experimental data were used as a dataset to construct a regression model, separating input and output data. A strong nonlinear regression model was defined and solved as shown in Eq 12:


$$Y=-19\mathrm{.57}x_{{}^{\text{1}}}-24\mathrm{.50}x_{\text{2}}-1096\mathrm{.25}x_{\text{3}}+55\mathrm{.99}x_{\text{1}}x_{\text{2}}+13760{\mathrm{.64}x}_{3}^{2}+52\mathrm{.93}$$ (12)

**Fig 12:**
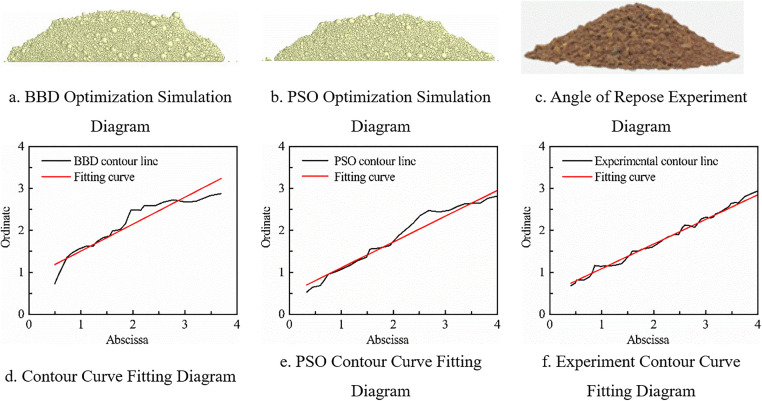
Comparison analysis of simulation and experiment.

**Fig 13:**
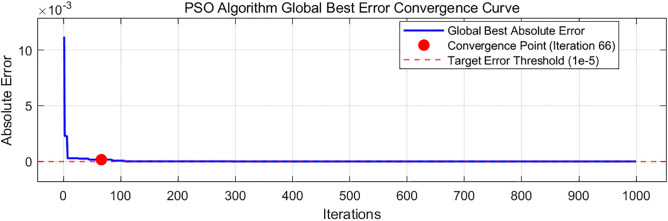
PSO algorithm global best error convergence curve.

Where *X*_1_, *X*_2_, and *X*_3_ represent the numerical values of *A*, *B*, and *G*, respectively. Nonlinear regression fitting was performed on the model, and the optimization objective function was defined. The target values were set as the experimental angles of repose of cohesive soil at different moisture contents, and the error values were calculated using Eq 9 with the goal of minimizing errors. The number of particles was 50, the maximum number of iterations was 200, and the parameter bounds for *A*, *B*, and *G* were set as follows: lower bounds of 0.27, 0.392, and 0.04; upper bounds of 0.39, 0.584, and 0.08. The Particle Swarm Optimization (PSO) algorithm was used to optimize the significant discrete element parameters of cohesive soil at different moisture contents. The optimization results are shown in Fig 12, and the angle of repose results are sorted in Table 11, which also compares the angles of repose optimized by the regression equation from BBD experiments. The global optimal error convergence curve of the Particle Swarm Optimization algorithm is shown in Fig 13. Convergence status: Converged; Convergence iteration number: 66; Stable error after convergence: 0.00042287 < 1e-5.

**Table 11:** PSO experimental results and error comparison analysis with BBD table.

Moisture content/%	*A*	*B*	*C*	Experimental value of angle of repose/°	PSO simulation measurement/°	PSO Error/%	BBD Error/%
0	0.344	0.578	0.063	30.83	30.638	0.62%	1.45%
5	0.336	0.566	0.064	31.56	31.889	1.04%	1.31%
10	0.390	0.539	0.065	31.97	32.039	0.22%	0.10%
15	0.282	0.584	0.068	33.15	32.434	2.16%	2.37%
20	0.336	0.546	0.074	37.13	37.136	0.02%	3.38%

It can be concluded from Table 11 that the PSO algorithm has smaller error values than the BBD algorithm in terms of angle of repose parameter optimization at moisture contents of 0%, 5%, 15% and 20%, but the BBD is more accurate for cohesive soil at 10% moisture content, with an error of only 0.12%.

The BBD algorithm constructs models based on discrete test points, with 17 test groups providing limited coverage in the 3D parameter space (A, B, G). This easily leads to “fitting blind zones” when water content changes cause migration of parameter-sensitive intervals. At 10% water content (BBD error: 0.10%, the lowest), the parameter sensitive interval exactly overlaps with BBD’s central test point (A = 0.33, B = 0.488, G = 0.06), ensuring high fitting accuracy. At 0%/15% water content, the sensitive interval migrates with water content (e.g., A’s interval shifts left to 0.28–0.34 at 0%, G’s interval shifts right to 0.068–0.072 at 15%). BBD’s fixed test points fail to cover the new intervals, leading to increased errors (1.45% at 0% water content).PSO achieves high-density search via continuous iteration of particles in the solution space (each particle is a potential solution). Even with sensitive interval migration, particles can quickly track new intervals through swarm collaboration (e.g., clustering around A = 0.28–0.34 and G = 0.063 at 0% water content), avoiding blind zone-induced errors. Thus, PSO errors at 0%/15% water content (0.62%/2.16%) are lower than BBD’s (1.45%/2.37%).

Water content variation essentially alters cohesive soil’s cohesion mechanism: van der Waals forces dominate at low water content (0%−5%), while liquid bridge forces prevail at high water content (15%−20%), transforming the parameter-repose angle relationship from “weak nonlinearity” to “strong nonlinearity.” BBD’s quadratic regression model (fixed form: Y = aA + bB + cG + dAB + eAG + fBG + gA² + hB² + iG²) cannot adapt to this mechanism shift. At high water content, parameter nonlinearity induced by liquid bridge forces (e.g., significant G² term impact with p = 0.0005 in Table 9) exceeds the model’s fitting capacity, causing deviations between simulated and actual values. PSO requires no preset model form and directly quantifies the nonlinear “parameter combination-repose angle error” relationship via particle fitness values. For example, at high water content, particles autonomously adjust G’s weight (e.g., G optimized to 0.074 at 20% water content, slightly higher than BBD’s 0.072), accurately matching liquid bridge force-dominated cohesion. Thus, PSO exhibits more significant error advantages in nonlinear intervals.

### 3.6 Convergence analysis of simulations with different particle mass gradients

**Fig 14:**
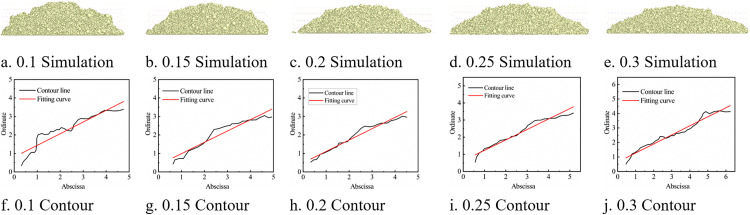
Simulation analysis diagrams of different particle masses.

To verify the correlation between the simulated angle of repose and the generated particle mass in discrete element simulations involving a large number of particles, the following experimental design is adopted. For the stacking simulation of cohesive soil, particle mass gradients are set as 0.1 kg, 0.15 kg, 0.2 kg, 0.25 kg, and 0.3 kg. The Hertz-Mindlin with JKR model is used, and the EDEM simulation parameters are those of cohesive soil at 10% water content. The simulation diagrams are shown in Fig 14, and the simulated stacking angle results are presented in Table 12.

**Table 12:** Simulated angle of repose results under different particle masses.

Particle mass/kg	0.1	0.15	0.2	0.25	0.3
**Angle of repose**/°	32.2	31.51	31.97	31.3	31.9

Key results: When the particle mass increases from 0.1 kg to 0.3 kg, the simulated angle of repose fluctuates from 31.3° to 32.2° (error: 2.8%); when the particle mass ≥ 0.15 kg, the angle of repose stabilizes between 31.3° and 31.97°, with an error of ≤2.14% between adjacent groups—confirming the results are independent of particle mass at this point.

Conclusion: 0.15 kg of particles is the convergence threshold for simulating the cohesive soil’s angle of repose. Beyond this mass, statistical randomness is suppressed, and the force chain network stabilizes.

### 3.7 Uniaxial compression test verification

To further verify the reliability of calibrated parameters, synchronous unconfined compression tests and discrete element (DEM) simulations were conducted on cohesive soil.

**Fig 15:**
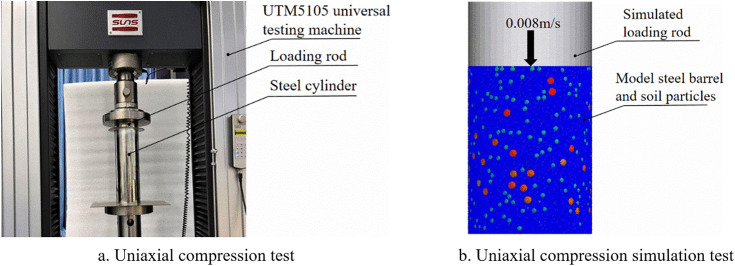
Uniaxial compression simulation test and physical test.

The physical tests employed a UTM5105 electronic universal testing machine, a steel cylinder (inner diameter 100 mm, height 300 mm), and a loading rod (diameter 99 mm) (Fig 15), with three water content levels (5%, 10%, 15%). In each test, 3 kg of cohesive soil was naturally poured into the steel cylinder; the testing machine pushed the loading rod downward at a constant speed of 0.008 m/s, and only post-loading axial stress-strain curves were recorded during compression. Tests were repeated 5 times for each water content level.

DEM simulations matched the physical test conditions, with consistent cohesive soil mass, water content, and loading rod descending speed. After the simulation, full-process axial pressure data were extracted via the DEM software’s post-processing module.

**Fig 16:**
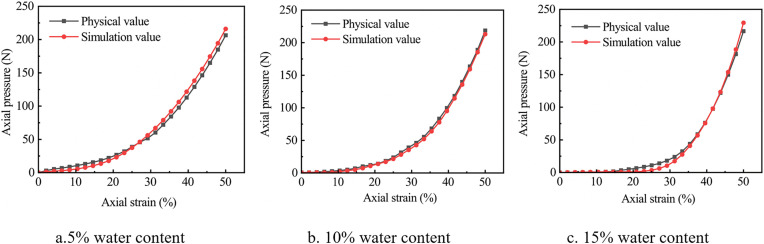
Comparison of simulated and physical compaction for cohesive soils at three water content levels.

Fig 16 compares axial stress-strain responses of cohesive soil under different water contents between physical compression tests and DEM simulations, reflecting soil compaction deformation. Their combination intuitively presents mechanical behavior changes during compaction.

Compaction of soil at all three water contents exhibits typical nonlinear stress growth: Low strain stage (0 ~ 10% axial strain): Stress increases gently. Large interparticle voids lead to deformation dominated by void filling, with weak particle contact and extrusion, requiring minimal compaction force. Medium strain stage (10% ~ 30% axial strain): Stress growth accelerates (steeper curve slope). Void filling progresses, particles contact and interlock, with friction and interparticle forces becoming dominant. High strain stage (30% ~ 50% axial strain): Stress surges sharply. Soil enters a “dense compaction” state with negligible voids; deformation requires overcoming particle elastic/plastic deformation and high friction, significantly increasing compaction stiffness.

Curves in each figure show excellent agreement: minimal numerical deviations and consistent trends across low-, medium-, and high-strain stages. This confirms the simulation model accurately captures the full-stage mechanical mechanism (void filling, particle contact, dense compaction) of cohesive soil compaction, with reliability and precision meeting engineering analysis requirements. Subsequent studies can replace some physical tests with simulations to reduce costs and improve efficiency.

Soil compaction characteristics vary significantly with water content: Low water content: Lowest stress in the low-strain stage and gentler stress growth in the high-strain stage, indicating low initial compaction resistance, easy compaction, but weak final bearing capacity. High water content: Higher stress in the low-strain stage and more pronounced stress surges in the high-strain stage, indicating high initial compaction resistance, difficult compaction, but strong final bearing capacity. Medium water content: Compaction characteristics between the above two.

This difference is essentially due to water content altering interparticle cohesion and lubricity, thereby affecting mechanical transmission during compaction.

Table 13 shows verification tests on gravelly soil with different water contents: the average error between simulated and measured axial pressures was 1.35 N, and the integral error of the integral difference relative to measured values was 5.1%. These results confirm that the proposed discrete element (DEM) simulation model, combined with parameter calibration, accurately describes the compression behavior of cohesive soil.

**Table 13:** Results of verification tests.

Moisture content	*A*	*B*	*G*	Physical integral	Simulation integral	Mean error/N	Integral error/%
5%	0.336	0.566	0.064	3030.11	3115.41	0.92	2.8
10%	0.390	0.539	0.065	2595.76	2461.58	1.35	5.1
15%	0.282	0.584	0.068	2010.93	1930.44	0.64	4.0

Overall, this not only verifies the model’s reliability in cohesive soil compaction analysis but also identifies water content as a key factor regulating compaction difficulty and bearing performance. In engineering, water content can be adjusted per actual needs to balance compaction construction cost and post-compaction bearing capacity, enabling a more efficient compaction process design.

### 3.8 Discussion

The potential limitations of the paper in capturing shear strength behavior are as follows: Single-dimensional information: The angle of repose only reflects the comprehensive mechanical effect under the steady state of particle accumulation. It cannot distinguish the individual contributions of the internal friction angle and cohesion, nor can it effectively characterize the stress-strain relationship during the shearing process. Environmental sensitivity: It is significantly affected by experimental methods (e.g., funnel height, particle feeding rate) and time effects (particle water absorption softening, attenuation of liquid bridge forces), which may lead to a disconnect between the calibrated parameters and actual shear working conditions. Difference in mechanical mechanisms: The angle of repose is determined by particle sliding equilibrium, while shear strength involves complex processes such as particle rearrangement and bond breakage. The dominant mechanical mechanisms of the two are different, which may cause deviations between simulation results and actual shear behavior. These limitations could serve as targets for future research.

Influence of mineral composition differences: Calibration parameters in this study are based on a specific southern cohesive soil (mineral type unspecified, presumably dominated by light clay minerals like kaolinite). Particle surface properties vary markedly among cohesive soils with different minerals: for example, montmorillonite-rich soils have strong crystal structure expansibility, resulting in much thicker interparticle water films and higher surface charge density than kaolinite soils, requiring substantial adjustments to core parameters such as JKR surface energy (characterizing adhesion) and static friction coefficient. The originally calibrated JKR surface energy range (0−25 J·m ⁻ ²) is incompatible with the high cohesion of montmorillonite soils; direct application underestimates simulated cohesion. Regulatory role of plastic index (PI): PI reflects soil clay content and water absorption capacity. While the PI of the soil in this study is unspecified, the calibrated parameters are strongly tied to particle gradation (e.g., 50% of particles are 0.315–0.63 mm). When extended to heavy clays with higher PI (e.g., PI > 30), the proportion of fine particles (<0.075mm) increases significantly, leading to more interparticle contacts and requiring a higher rolling friction coefficient to restrain particle sliding. In contrast, for sandy clays with lower PI (PI < 10) and a high proportion of coarse particles, the originally calibrated static friction coefficient may be excessively large, potentially overestimating the simulated angle of repose. Lack of shear strength parameters: The original calibration only correlates cohesion and internal friction angle via the angle of repose, failing to distinguish their individual contributions. However, shear failure of other cohesive soils may be dominated by particle rearrangement and bond breakage.

Limitations of calibrated parameters in simulating dynamic processes: Statistically calibrated parameters only fit particles’ mechanical behavior during low-speed contact and steady-state force chain balance, with notable limitations in direct application to dynamic processes. Dynamic processes include transient inertial effects, stress wave propagation, and cohesive mechanism dynamic evolution—features unaddressed by static parameters, reducing simulation accuracy. A key flaw is the dynamic friction coefficient: statically screened yet calibrated at low speeds (0.1 ~ 0.5 m/s). In high-speed impact, particle sliding speed hits 1 ~ 10 m/s; high strain rates cause local temperature rise via surface friction. Neglected in static calibration, this temperature effect leads to friction resistance calculation deviations. Static parameters only suit low-speed steady-state scenarios. Inherent mechanical property differences between dynamic and static scenarios cause notable errors in direct application. Adapting to dynamic processes requires dynamic parameter correction, supplementary dynamic calibration, and coupled dynamic constitutive optimization. This aligns with contemporary discrete element calibration technology progress—“scenario-specific parameter optimization”—where calibrated parameters must closely match the target working condition’s mechanical mechanism, instead of a single universal static calibration.

Sensitivity of Key Calibrated Parameters (A, B, G) to Particle Gradation Changes. Based on Table 6’s significance analysis (characterized by F-values), the sensitivity of parameters to particle gradation changes is clear: JKR surface energy (G, contribution 63.5%, F = 63.5, p < 0.01) is highly sensitive; soil-soil static friction coefficient (B, 8.44%, F = 8.44, p < 0.05) is moderately sensitive; soil-soil restitution coefficient (A, 8.44%, F = 8.44, p < 0.05) is low sensitive. The core lies in gradation affecting parameters’ impact on the angle of repose by altering particle contact morphology: when fine particle content increases significantly, only A can be fine-tuned for adaptation, while B and G require recalibration. A (restitution coefficient): Low sensitivity. Dominated by coarse particles in the original gradation, A only affects collision energy consumption without changing the steady-state force chain. After fine particle increase, collision mode changes but energy consumption rises slightly, enabling adaptation via linear fine-tuning. B (static friction coefficient): Moderate sensitivity. The original gradation relies on coarse particle edge friction; fine particles form a “lubricating layer” to weaken this effect. Original parameters overestimate sliding resistance, requiring recalibration to F ≥ 8.0 (p < 0.05). G (JKR surface energy): High sensitivity, with a contribution 7.5 times that of A and B, dominating adhesion. After fine particle increase, the adhesion mechanism changes; the original G underestimates adhesion, leading to aggregate dispersion and a low angle of repose. Recalibration via the PSO algorithm for the new gradation is needed to match the original contribution ratio.

This study’s findings integrate with the latest research on soil-water interaction and microstructure evolution. Su et al [29] found SWRC dominated by dry density at low suction (<715 kPa) and fine-particle microstructure at high suction, with JKR surface energy linked to water film-suction coupling and liquid bridge stability—laying the foundation for surface energy-SWRC empirical relationships. Zheng & Baudet [30] and Zheng et al [31] revealed pore evolution during one-dimensional clay compression via multi-scale techniques, clarifying microstructure-macro parameter correlation and inspiring a compressible aggregate model for DEM parameter inverse optimization. Li et al [32] proposed organic soil shear behavior mechanisms, and this study’s JKR surface energy and friction parameters can be extended to shear simulation for micro-parameter characterization. SEAGJ [33] noted that cohesive soil friction angle is coupled with water content/gradation, with micro-macro nonlinear mapping providing a basis for prediction models and engineering parameter estimation.

Integrating these findings endows DEM parameters with clear physical significance, enhancing the model’s applicability and predictability; future work will establish an integrated framework for a full-chain closed loop.

Without experimental validation for the southern cohesive soil in this study, the shear modulus (1 × 10⁶ Pa) and Poisson’s ratio (0.38) are directly adopted from literature, introducing potential uncertainties: first, they affect the effective elastic modulus of the JKR model, potentially causing deviations between simulated and actual repose angles; second, mismatched shear modulus may result in “overly stiff” or “overly soft” soil simulations (prone to resistance errors in dynamic scenarios), while Poisson’s ratio deviations induce contact stress distribution errors; third, both parameters dynamically change with water content, and fixed literature values ignore this characteristic, undermining the correlation logic of “water content - parameters - macro response.”

## 4 Conclusions

(1) Construction of the moisture content-angle of repose model for cohesive soil: By preparing five cohesive soil samples with different moisture contents (0%, 5%, 10%, 15%, 20%), measuring their density and other intrinsic parameters, using the vibration sieving method to determine particle size distribution, and conducting angle of repose experiments with a funnel device, it was concluded that the angle of repose increases with increasing moisture content, and a polynomial regression equation model between the two was established. The coefficient of determination R² of the model is 0.9995.
(2) Establishment of the angle of repose-discrete element key parameter model for cohesive soil: Relevant parameters were obtained through static and dynamic friction coefficient measurement experiments, more parameters were selected using the GEMM library, and discrete element EDEM modeling was performed. The Plackett-Burman significance test analysis was used to screen factors significantly influencing the angle of repose (JKR surface energy, soil-soil restitution coefficient, soil-soil static friction coefficient). Through climbing experiments and response surface analysis, it was clarified that JKR surface energy, soil-soil restitution coefficient, and soil-soil static friction coefficient have a positive correlation with the angle of repose, and the interaction between JKR surface energy and soil-soil restitution coefficient/soil-soil static friction coefficient is similar, providing a basis for parameter optimization.
(3) Construction and optimization of the moisture content-discrete element key parameter model for cohesive soil: Linking the previous two models to construct a new model, the key discrete element parameters of cohesive soil at different moisture contents were optimized using the conventional BBD method. A single-objective optimization algorithm (PSO) was then used for comparative experiments. The results show that the PSO algorithm has smaller angle of repose parameter optimization errors than the BBD algorithm at moisture contents of 0%, 5%, 15%, and 20%, while the BBD algorithm is more accurate at 10% moisture content. The key parameters—soil-soil restitution coefficient, soil-soil static friction coefficient, and JKR surface energy—for the five moisture contents are selected as shown in Table 11.

### Suggestions

Based on the core conclusions of DEM parameter calibration for cohesive soil under multiple water contents in this study, concise and feasible suggestions are proposed from three aspects—engineering practice, follow-up research, and method reuse—to maximize the research’s application value. For the parameter calibration process, the workflow of “basic parameter determination (sieving/ inclined plane method/repose angle test) – PBD screening of significant parameters (focus on G, A, B) – ramp test for boundary definition – BBD/PSO optimization” is prioritized, and PSO is recommended for multi-water content scenarios (error < 2.2%); the recommended parameter ranges are: JKR surface energy (G) 0.04–0.08 J·m ⁻ ², soil-soil static friction coefficient (B) 0.392–0.584, and restitution coefficient (A) 0.27–0.39, with direct application of the wide ranges in the GEMM database (e.g., original G range: 0–25 J·m ⁻ ²) to be avoided. For follow-up research, supplementary tests should verify the shear modulus/Poisson’s ratio (currently adopted from literature) through resonant column tests and establish the “water content - water film thickness - G” relationship using low-temperature scanning electron microscopy (LT-SEM) to enhance the physical significance of parameters; in terms of scenario expansion, loader loading tests should be combined to build a “static parameters - dynamic resistance” model and modify the G value under high strain rates; regarding error control, errors should be corrected in the priority order of “G first, then B, and finally A”, with the static repose angle error required to be < 2.5% and the dynamic resistance error < 8%.

## Supporting information

S1 File Particle size distribution data. (XLSX)

S2 File Plackett-Burman test data processing. (DOCX)

S3 File Steepest ascent experiment data. (XLSX)

S4 File Box-Behnken test data processing. (XLSX)
