# Supplementary material for: Calibration of discrete element parameters for cohesive soils at different moisture contents
Source: PLoS One. 2026 Feb 9;21(2):e0340462. doi: 10.1371/journal.pone.0340462 (PMC12885266; doi:10.1371/journal.pone.0340462)
Supplement: S2 File — (DOCX) [file pone.0340462.s002.docx]

| **Serial Number** | **Symbols** | | | | | | | **Angle of Repose/°** |
| --- | --- | --- | --- | --- | --- | --- | --- | --- |
|  | ***A*** | ***B*** | ***C*** | ***D*** | ***E*** | ***F*** | ***G*** |  |
| 1 | -1 | 1 | -1 | 1 | 1 | -1 | 1 | 38.7 |
| 2 | -1 | 1 | 1 | -1 | 1 | 1 | 1 | 31.74 |
| 3 | -1 | -1 | 1 | -1 | 1 | 1 | -1 | 13.94 |
| 4 | 1 | -1 | 1 | 1 | 1 | -1 | -1 | 18.36 |
| 5 | 1 | -1 | 1 | 1 | -1 | 1 | 1 | 85.29 |
| 6 | -1 | -1 | -1 | -1 | -1 | -1 | -1 | 14.93 |
| 7 | -1 | -1 | -1 | 1 | -1 | 1 | 1 | 76.73 |
| 8 | -1 | 1 | 1 | 1 | -1 | -1 | -1 | 17.47 |
| 9 | 1 | 1 | -1 | 1 | 1 | 1 | -1 | 22.52 |
| 10 | 1 | 1 | 1 | -1 | -1 | -1 | 1 | 56.06 |
| 11 | 1 | -1 | -1 | -1 | 1 | -1 | 1 | 74.87 |
| 12 | 1 | 1 | -1 | -1 | -1 | 1 | -1 | 26.68 |

PB
